# Rare copy number variations containing genes involved in RASopathies: deletion of *SHOC2* and duplication of *PTPN11*

**DOI:** 10.1186/1755-8166-7-28

**Published:** 2014-04-16

**Authors:** Jin-Lan Chen, Xin Zhu, Tian-Li Zhao, Jian Wang, Yi-Feng Yang, Zhi-Ping Tan

**Affiliations:** 1 Department of Cardiothoracic Surgery, the 2nd Xiangya Hospital of Central South University, Changsha, China; 2 Clinical Center for Gene Diagnosis and Therapy of State Key Laboratory of Medical Genetics, the Second Xiangya Hospital, Central South University, Changsha, China; 3 Department of Gynecology and Obstetrics, Xiangya Hospital of Central South University, Changsha, China

**Keywords:** Noonan syndrome, RASopathy, *SHOC2*, *PTPN11*, 12q24 duplication, 10q25.2 deletion, Copy number variation, CNV, Congenital heart defect

## Abstract

**Background:**

RASopathies are a group of disorders related to Noonan syndrome that with dysregulated RAS-mitogen-activated protein kinase (MAPK) signaling pathway. Noonan syndrome (NS, OMIM# 163950) is a both phenotypically and genotypically variable disorder. We and other researchers have demonstrated that copy number variations underlie a small percentage of patients with RASopathies.

**Results:**

In a cohort of 12 clinically characterized patients with congenital heart defect (CHD) and features suggestive of Noonan syndrome or Noonan like syndrome without known causative gene mutation, we performed an Illumina SNP-array analysis to identify the pathogenic copy number variations (Human660W-Quad Chip, Beadstation Scanner and GenomeStudio V2011 software).

We identifed two rare copy number variations harboring genes involved in RAS- MAPK signaling pathway of RASopathy. One is a 24 Mb duplication of 12q24.1-24.3 containing *PTPN11* and the other is a 183 kb deletion of 10q25.2 including *SHOC2*. The SNP-array results were further validated by quantitative PCR (qPCR). This is might be the first report suggesting that haploinsufficiency of *SHOC2* can result in a RASopathy-like phenotype.

**Conclusions:**

Our findings provide additional support that copy number variations containing disease-causing genes of RAS/MAPK pathway play a minor role in RASopathies or related disorders. We recommend the use of microarrays in Noonan syndrome like patients without identified mutations in the causative genes.

## Background

RASopathies define a group of disorders clinically related to Noonan syndrome, which are caused by germline mutations in genes encoding components in the RAS-mitogen-activated protein kinase (MAPK) signaling pathway [1-3]. Over the past decade, several genes including *PTPN11* (50% of patients with NS), *SOS1* (5-10%), *RAF1* (5-10%), *KRAS* (2%), *BRAF* (2%), *NRAS*, *MEK1*, *SHOC2*, *CBL* and *RIT1* have been identified in 70-75% of affected cases [4-9]. Recent studies using high-resolution chromosomal microarrays (SNP-array or CGH-array) suggest that copy number variations involved aforementioned genes. Duplications of 12q24 encompassing *PTPN11* have been identified as an uncommon cause of NS [10-12]. In 2012, we reported the first case suggestive of Noonan syndrome carrying a microduplication of *RAF1*[13]. In 2013, haploinsufficiency of *MAP2K2*/*MEK2* was proposed as a novel mechanism for the pathogenesis of RASopathies [14].

In this study, we described two additional patients with distinct phenotype. A 183 kb deletion of 10q25.2 including *SHOC2* and a 24 Mb duplication of 12q24.1-24.3 containing *PTPN11* were identified in a sporadic case. Our results add new insights that copy number variations play an import role in the etiology of NS patients without known causative gene mutations.

## Methods

This study was approved by the Review Board of the Second Xiangya Hospital of the Central South University for research involving human subjects. Written consent to publish photographs of all the patients as well as clinical data were obtained from the parents of the patients.

### Patients

#### Patient 1

**Figure 1:**
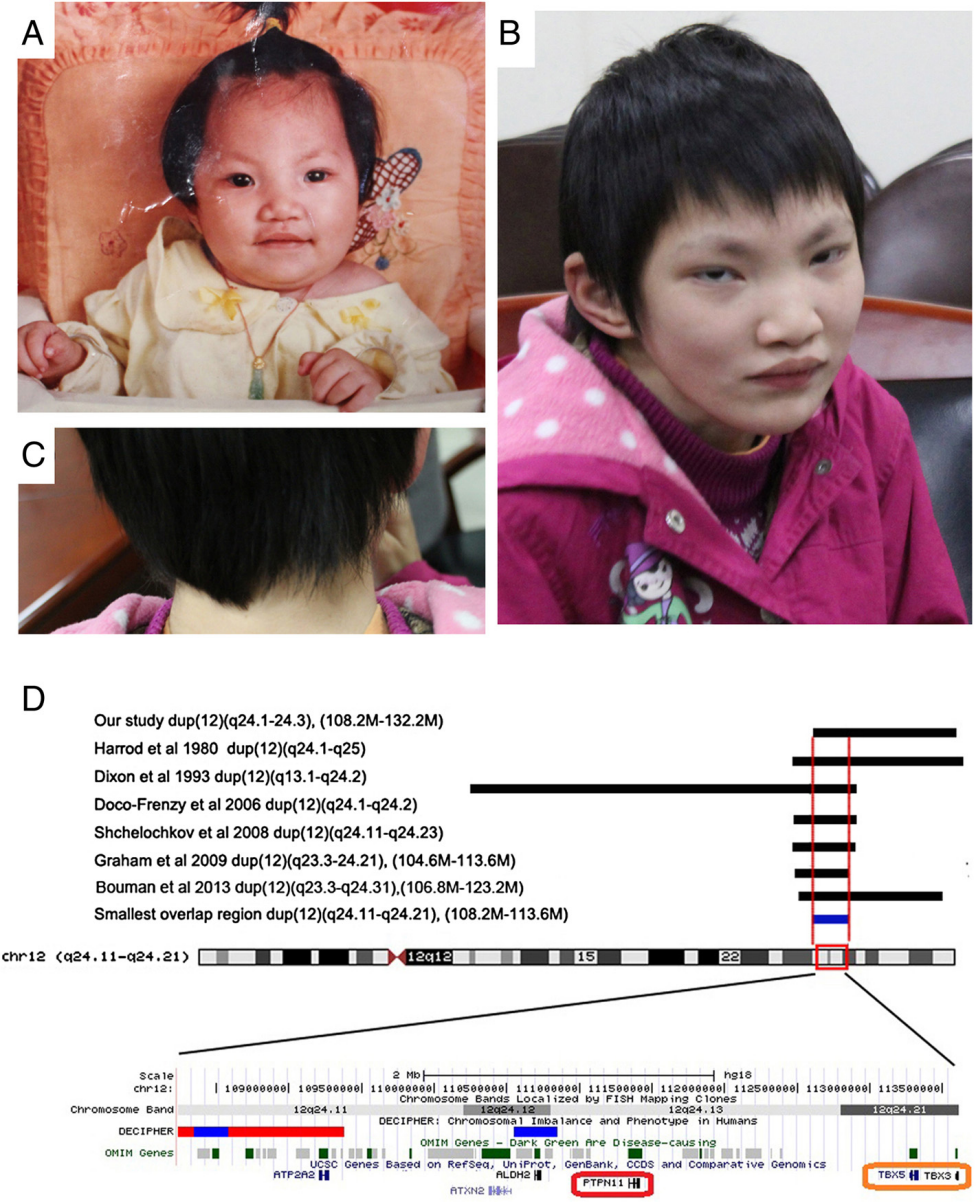
**Craniofacial features of patient 1(A-D). (A) **one year old, **(B)** and **(C)** 14 years old, Note the distinctive facial features, downward slanting palpebral fissures, a large mouth with downturned corners, a short webbing neck and low posterior hairline. **(D)** Schematic of the duplications in previously report showing the relative positions of *PTPN11* and *TBX5*. The upper seven black bars represent the reported duplications. The smallest overlap region (108.2 Mb-113.6 Mb) is indicated in blue bar. Genomic data have been converted to Hg19.

Patient 1 was first seen in our Department of Cardiothoracic Surgery at the age of 14 years because of heart defect, ventricular septal defect (VSD). Further clinical evaluation revealed distinctive facial features including downward slanting palpebral fissures, a large mouth with downturned corners, a short webbing neck and low posterior hairline (Figure 1). Other abnormalities included widely spaced nipples, thoracic scoliosis to right and clinobrachydactyly. The proband is the only child of healthy non-consanguineous parent with normal family history. The patient was born at term of gestation after an uncomplicated pregnancy and delivery. She was born with a weight of 3,200 g (50th centile) and a height of 49 cm (50th centile). Pectus excavatum became more obvious since six years old. She had developmental delay, crawling at age of two and walking at three years old. She had a speech delay and can only speak single words. At age of 14 years, she was very thin with a weight of 35 kg (3rd centile), and a length of 120 cm (3rd centile). She had severe mental retardation which needs special education.

#### Patient 2

**Figure 2:**
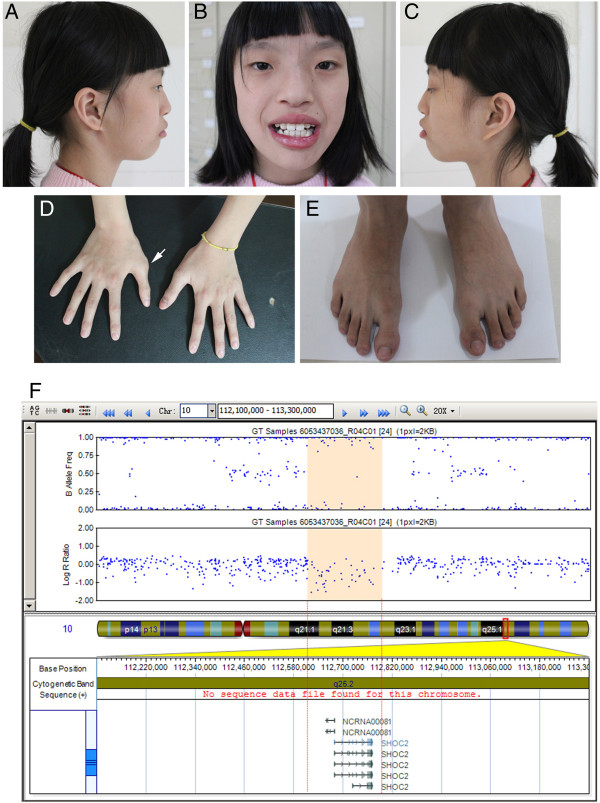
**Clinical features of patient 2(A-E).** Lateral **(A, C)** and frontal **(B)** view of the patient. The patient has facial asymmetry, sparse eyebrow, a large nasal tip and a long midface with a large mouth and crowding teeth. **(D)** Hands and **(E)** feet of the patient. Arrow indicates the surgically removed preaxial polydactyly. **(F)** Human 660w-Quad SNP-array analysis of the 10q25.2 deletion including *SHOC2* in the Patient 2. SNP-array shows a 0.18 Mb deletion in 10q25.2 (chr10: 112611576-112795021/Hg19). Log R ratio and B allele frequency are showed in the upper panel; deleted genes are showed in the lower panel.

Patient 2 was an 11-year-old girl who had complex CHD and entered our Department of Cardiothoracic Surgery for surgical correction. She was the first child of a healthy non-consanguineous parent. She was born at full term with a weight of 3,150 g (50th centile) and a height of 50 cm (50th centile). She had severe cyanotic heart disease (double outlet right ventricle, DORV and a large 20 mm VSD). Preaxial ploydactyly was observed in the right hand (Figure 2) and was surgically removed at the age of two years. Clinical evaluation revealed dysmorphic features, including facial asymmetry, microcephaly with sparse eyebrow, a large nasal tip, a long midface with a large mouth and crowding teeth. The last examination was performed at the age of 12 years. She weighed 30 kg (10th centile) with a height of 150 cm (50th centile). Neurological assessment showed normal intelligence, however with some behavioral disorders such as hyperactivity.

### DNA extraction

The parents of the patient gave written informed consent and genomic DNA was prepared from peripheral blood of the patient and his parents. Genomic DNA was prepared using a DNeasy Blood & Tissue Kit (Qiagen, Valencia, CA) on the QIAcube automated DNA extraction robot (Qiagen, Hiden, Germany).

### Mutation sequencing

The entire coding regions, including the flanking intronic sequences of aforementioned 10 genes associated with NS or Noonan like syndrome, were amplified with polymerase chain reaction (PCR; primer sequences will be provided upon requests). Sequences of the PCR products were determined using the ABI 3100 Genetic Analyzer (ABI, Foster City, CA) as previously described [15,16].

### SNP array analysis

The Human660W-Quad Chip (Illumina Inc, San Diego, USA) and the Illumina BeadScan genotyping system (Beadstation Scanner) were employed to obtain the signal intensities of probes (SNP) following the manufacturer’s instructions. The GenomeStudio V2011 software was used to analyze the genotypes (human genome build 37/Hg19) and evaluate the experimental quality. The call rates of the samples are greater than 99.0%.

### Quantitative PCR validation

To validate variable copy numbers, real-time quantitative PCR (qPCR) were performed using the 7500 Fast Real-Time PCR systems (Applied Biosystems, Foster City, California). Two primer sets were designed within the boundaries of the CNV region. Primer pairs were designed by an online tool (PrimerQuest, IDT) (http://www.idtdna.com/Primerquest/Home/Index). PCR reactions were prepared with the SYBR Premix Ex Taq II PCR reagent kit (TaKaRa Bio, Dalian, China) according to the manufacturer’s protocol. Amplification efficiencies were identical and the relative copy number was calculated with the 2^-ΔΔCt^ method.

## Results

We report here two additional patients with distinct phenotypes suggesting RASopathies. Both patients have a characteristic facial feature, congenital heart defects, skeletal malformations and ectodermal abnormalities (*e.g.* sparse hair and sparse eyebrow). Bidirectional sequencing of the aforementioned causative genes did not identify any pathogenic mutations. We hypothesized that chromosome structural variations might underlie both cases and employed SNP-array to identify such possible changes (Illumina Human660W-Quad) [17]. To identify the *de novo* possible pathogenic CNVs, we examined genomic DNA samples from parent-patient trios and identified a 24 Mb duplication of 12q24.1-24.3 containing *PTPN11* in Patient 1 (chr12:108235593-132289191/Hg19) (Figure 1, Additional file 1: Figure S1), and a 183 kb deletion of 10q25.2 including *SHOC2* in Patient 2 (chr10: 112611576-112795021/Hg19) (Figure 2). Parents of both patients did not carry the related genomic lesions.

## Discussion

In this study, we report on two patients with molecularly confirmed diagnosis of RASopathy carrying different chromosomal imbalances, but all contained a critical gene related to the RAS/MAPK signaling pathway.

Patient 1 (12q24.1-24.3 duplication) has some typical features of NS, such as webbed neck, short stature, severe developmental delay and pectus excavatum. A congenital heart defect (ventricular septal defect, VSD) was identified by echocardiogram and was surgically repaired in our Department of Cardiothoracic Surgery. To date, Ten cases (including this study) have been reported to carry similar 12q24 duplication encompassing *PTPN11*[10-12,18]. In order to reveal possible candidate genes involved in the pathogenesis of 12q24 duplication, we aligned the chromosomal duplication region in reported individuals. Our study of Patient 1 further narrowed the critical region to a 5.4 Mb interval (108.2 Mb-113.6 Mb) (Figure 1A). About 55 genes, including *PTPN11* and *TBX5*, are located in this duplication region. Given that overexpression of *TBX5* has been associated with cardiac development, we could not exclude the possibility that the cardiac defect (VSD) found in Patient 1 might be a consequence of *TBX5* duplication. Also some phenotypes that might not be associated with RASopathies could be explained by the contribution of the other critical genes in the duplicated region, i.e. severe developmental disability and ocular anomalies could be caused by *UBE3B*[19], and brachydactyly could be explained by *TRVP4*[20].

Activating gain-of-function mutations in *SHOC2* cause NS with loose anagen hair. Interestingly, only one recurrent *SHOC2* mutation (p.S2G) has been described [21]. A high frequency of short stature (100%) and intellectual disability (84%) was observed in NS with loose anagen hair [21]. However, Patient 2 (10q25.2 deletion) had normal stature and normal intelligence, though with unique phenotype such as preaxial polydactyly of the right hand (Figure 1B). We hypothesize that this novel 10q25.2 deletion might represent a previously unrecognized disorder. Beside *SHOC2*, *BBIP1* (also known as *NCRNA00081*/*BBIP10*) is also mapped in the deleted region. *BBIP1* was recently shown to cause a recessive type of Bardet-Biedl syndrome with retinitis pigmentosa, obesity, kidney failure, cognitive disability and brachydactyly [22], phenotypes that are obviously different from our patient. We determined the sequences of the entire coding and flanking intronic regions of *BBIP1* and failed to identify a mutation in the existing allele of *BBIP1* (primer pairs are included in the Additional file 2: Table S1). Therefore *BBIP1* mutations might not underlie this disorder.

CHD is observed in 50-75% of patients with syndromes of the RAS-MAPK signaling pathway [23,24]. Pulmonary valve stenosis (PS) and atrial septum defect (ASD) are frequently observed in patients with NS. The most commonly cardiac defects in patients with *SHOC2* mutation are dysplasia of the mitral valve and septal defects [21]. In our study, patient 2 (deletion of *SHOC2*) had DORV, which was seldom reported in Noonan syndrome or Noonan-like syndrome [25].

Despite extensive efforts to identify disease-causing genes in NS and NS-like disorders, approximately 25-30% of patients remain genetically undiagnosed. It highlights multiple pathogenic mechanisms underlying RASopathy. The majority of the mutations identified in the RASopathies are gain-of-function which resulted in increased RAS/MAPK signaling. However, loss-of-function mutations are also reported to cause LEOPARD syndrome [26]. Previous studies have demonstrated that copy number variations containing disease-causing genes play a minor role in RASopathies [12,13]. Furthermore, happloinsufficency of *MAP2K2*/*MEK2* was recently reported be a novel mechanism for the etiology of RASopathies [14]. In line with these findings, our study that deletion of *SHOC2* provides additional evidence that microdeletion containing one of the components of the RAS/MAPK pathway could result in Noonan syndrome related disorder.

## Conclusions

We describe pathogenic copy number variations in two individuals, a 24 Mb duplication of 12q24 containing *PTPN11* resulted in apparent Noonan syndrome, while the 183 kb deletion of 10q25.2 including *SHOC2* contributed to atypical Noonan-like syndrome. To our knowledge, deletion of *SHOC2* might be the first report in RASopathies. Our study also provides additional support that copy number variations containing disease-causing genes of RAS/MAPK pathway play a minor role in RASopathies.

## Abbreviations

CNV: Copy number variation; CHD: Congenital heart defects; SNP: Single nucleotide polymorphism; PCR: Polymerase chain reaction; CT: Computed tomography; OMIM: Online Mendelian Inheritance in Man; Hg19: Human genome 19; ASD: Atrial septal defect; DORV: Double outlet right ventricle; DGV: Database of Genomic Variants; NS: Noonan syndrome; MAPK: Mitogen-activated protein kinase.

## Competing interests

The authors declare that they have no competing interests.

## Authors’ contributions

CJL completed the laboratory work, and co-wrote the manuscript with TZP; TZP supervised the laboratory work; LZM and ZTL reviewed all laboratory results and patient data and YYF reviewed all the clinical data; WJ supervised the design of the SNP-array. All authors read and approved the final manuscript.

## Supplementary Material

Additional file 1: Figure S1 SNP-array result of Patient 1 showing a 24 Mb duplication of 12q24 containing *PTPN11* (chr12:108235593-132289191/Hg19). Click here for file

Additional file 2: Table S1 Primer pairs for mutation screening of *BBIP1* in Patient 2. Click here for file
