# Supplementary figures and images for: Rare copy number variations containing genes involved in RASopathies: deletion of SHOC2 and duplication of PTPN11
Source: Mol Cytogenet. 2014 Apr 16;7:28. doi: 10.1186/1755-8166-7-28 (PMC4031927; doi:10.1186/1755-8166-7-28)

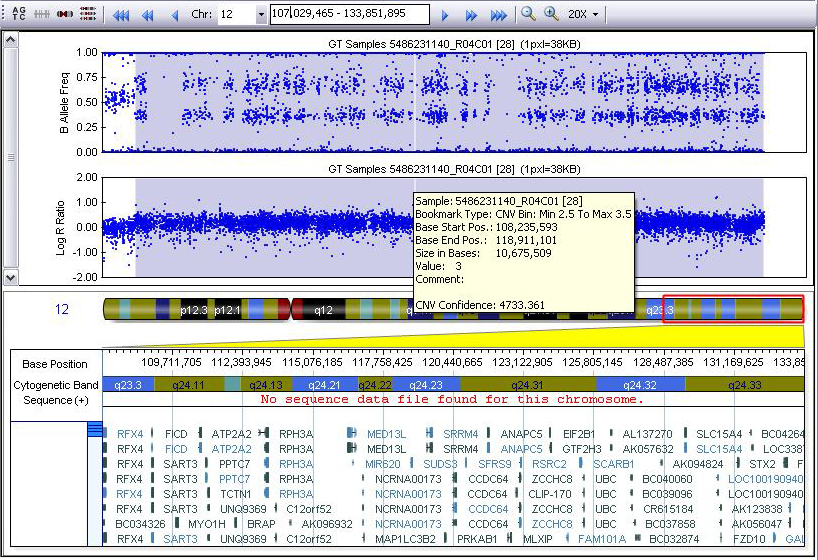

Supplement: Additional file 1: Figure S1 — SNP-array result of Patient 1 showing a 24 Mb duplication of 12q24 containing PTPN11 (chr12:108235593-132289191/Hg19). [file 1755-8166-7-28-S1.jpeg]
